# Adult hepatic cavernous hemangioma with highly elevated α-fetoprotein: A case report and review of the literature

**DOI:** 10.3892/ol.2014.2769

**Published:** 2014-12-04

**Authors:** HUAN-YU WANG, LIANG ZHANG, JIAN WU, ZI-JIAN ZHANG, BAO-GANG PENG, LI-JIAN LIANG, QI ZHOU

**Affiliations:** 1 Department of Hepatobiliary Surgery, The First Affiliated Hospital of Sun Yat-Sen University, Guangzhou, Guangdong 510080, P.R. China; 2 Diagnostic Imaging and Interventional Radiology Department, Cancer Center, Sun Yat-Sen University, Guangzhou, Guangdong 510080, P.R. China

**Keywords:** hepatic cavernous hemangioma, α-fetoprotein, cancer stem cell

## Abstract

A 47-year-old male presented with a six-month history of fatigue and a four-month history of alanine and aspartate aminopherase elevation. Laboratory examination revealed that the serum α-fetoprotein (AFP) level was 371.51 μg/l (normal range, 0–20 μg/l), and a computed tomography scan revealed a hypodense lesion in the left hepatic lobe. During laparotomy, a dark red-colored soft tumor (1.5×1.7 cm in diameter) was found in segment eight of the liver. Intra-operative pathology and post-operative histopathology examinations revealed that the tumor was a hepatic cavernous hemangioma. The serum AFP level was decreased to 24.45 μg/l by the second post-operative week. The literature was searched and only three similar cases were found. A brief review of this rare disease entity was produced, which attempted to explain this appearance reasonably.

## Introduction

Among the hepatic vascular tumors, cavernous hemangioma and infantile hemangioendothelioma are benign, while epithelioid hemangioendothelioma and angiosarcoma are malignant. Cavernous hemangioma occurs at all ages, but most frequently in adults, while infantile hemangioendothelioma occurs between birth and 21 years of age (1). α-fetoprotein (AFP) is a fetal-specific glycoprotein produced by the fetal liver. Usually, the AFP serum concentration falls rapidly subsequent to birth, while normal adult levels are usually achieved by the age of eight to 12 months. The normal range of AFP for adults and children is variously reported as <50, <10 and <5 μg/l (2,3). Clinically, AFP is one of the indicators that aids in the diagnosis of hepatocellular carcinoma (HCC), particularly for the patients with chronic liver diseases (3–5). There are numerous studies that have reported a higher level of AFP serum exhibiting an increased association with a poor prognosis for patients with HCC (4,6). There are numerous other diseases, excluding HCC, that are also associated with an increased serum AFP level, including fulminant hepatitis, hepatic cirrhosis, gastric cancer and endodermal sinus tumors of the testes, ovaries and extragonadal sites (7–13). As one of the benign neoplasms, the AFP level of hepatic cavernous hemangioma patients is not usually outside the normal range. The present study reports the case of hepatic cavernous hemangioma with a highly elevated AFP level in a 47-year-old male. Written informed consent was obtained from the patient.

## Case report

### Patient history

A 47-year-old male was admitted to the Department of Hepatobiliary Surgery (The First Affiliated Hospital of Sun Yat-Sen University, Guangzhou, Guangdong, China) on 14th February 2013 with a six-month history of fatigue and a four-month history of elevated alanine and aspartate aminopherase levels according to colorimetric assay. The patient possessed a 20-year history of chronic hepatitis B with no medical control. In January 2013, the patient was admitted to Dongguan Kanghua Hospital (Dongguan, Guangdong, China) with a five-month history of fatigue.

### Examination

During his hospitalization at Dongguan Kanghua Hospital, abdominal computed tomography (CT) scans revealed a tumor located in the right liver, with good enhancement in the arterial and portal venous phases, and multiple cysts in the liver. Magnetic resonance imaging (MRI) revealed a tumor with good enhancement. A diagnosis of hemangioma was considered, but hepatocellular carcinoma could not be excluded. On 4th February, abdominal sonography in the outpatient clinic of The First Affiliated Hospital of Sun Yat-Sen University revealed a solid nodule located in segment eight of the liver, multiple cysts in segments 2, 4 and 5 of the liver, calcification in segment six of the liver and multiple gallbladder polyps. The serum level of AFP was 371.51 μg/l (normal range, 0–20 μg/l). During physical examination, no ascites were detected and the abdomen was tender on palpation. The results of the laboratory tests performed during hospitalization are shown in Table I. Serum levels of tumor markers, including AFP, carcinoembryonic antigen (CEA) and carbohydrate antigen (CA)-199 were out of the normal range (Table II).

**Figure 1:**
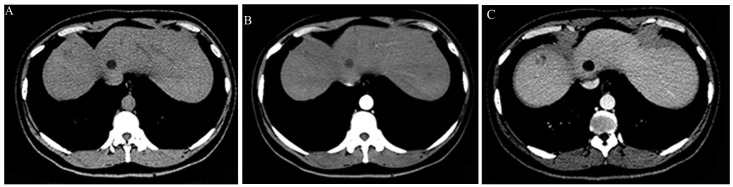
Computed tomography scans revealing (A) a 1.3-cm well-defined tumor in segment eight of the liver. The tumor presents (B) punctate enhancement in the early phase while (C) the enhancement becomes stronger in the delayed phase.

**Figure 2:**
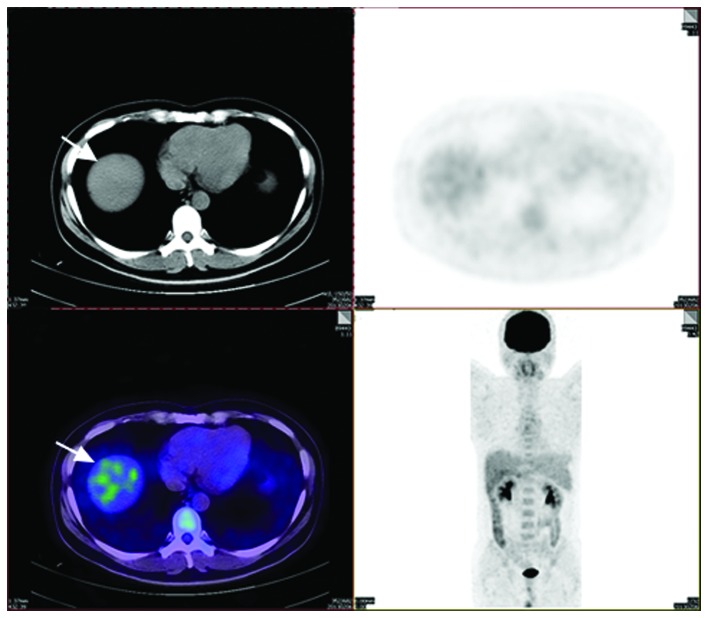
Positron emission tomography revealing a tumor (arrow) of low intensity with a mildly active metabolism in segment eight of the liver.

CT scans revealed a 1.3-cm well-defined tumor located in segment eight of the liver (Fig. 1), with good enhancement in the arterial and portal venous phases, multiple hypodense cysts in the liver and calcified lymph nodes prior to segment five in the abdominal cavity. The positron emission tomography (PET) study revealed a tumor of low intensity (Fig. 2), multiple hypodense cysts and multiple gallbladder polyps. Due to the age of the patient, the presence of a hepatic hypodense lesion resembling a tumor and the high tumor marker levels, the differential diagnosis comprised hepatocellular carcinoma, focal nodular hyperplasia and hemangioma. To exclude hepatocellular carcinoma, a typical hepatectomy was recommended to remove segment eight of the liver and gall bladder.

### Treatment

**Figure 3:**
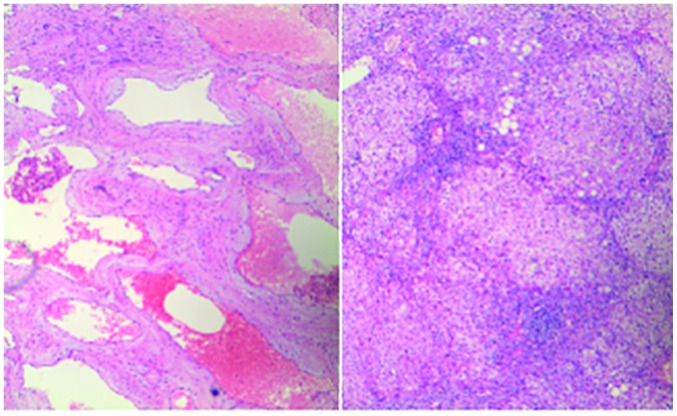
Histopathology examinations revealing that the tumor was a cavernous hemangioma of the liver (hematoxylin and eosin stain; magnification, ×40).

Exploratory laparotomy was performed on 18th February 2013, and a dark red-colored soft tumor (1.7×1.5 cm in diameter) in segment eight of the liver was revealed. Intra-operative ultrasound examination was performed, which identified no other lesions in the remaining sections of the liver. Intra-operative pathology and post-operative histopathology examinations revealed that the tumor was a cavernous hemangioma of the liver (Fig. 3). The serum AFP level had decreased to 111 μg/l by post-operative day seven and to 24.45 μg/l by post-operative week two.

## Discussion

Cavernous hemangioma is the most common hepatic vascular tumor in adults (1). However, the carcinoma biomarkers of hemangioma are usually within the normal range (2,14,15). The abdominal ultrasound examination and CT scan performed on the present patient revealed a localized hepatic nodule with the characteristics of hepatic hemangioma, while the highly elevated AFP and CEA levels suggested HCC. However, the intra-operative pathology and post-operative histopathology examinations revealed a liver cavernous hemangioma. The literature was searched and only three cases were found, one in English and the other two in Chinese (14,15). All the patients in the previously reported cases were Chinese, consisting of one male and two females. All three cases were diagnosed following a routine health examination and were negative for the hepatitis B surface antigen. Their pre-operative AFP levels were 1890, 784 and 224 μg/l, respectively, and returned to normal levels (0–20 μg/l) within four weeks of the surgical removal of the tumor. The tumors were not located in the same segments, but the three reported cases were all >6.0 cm in diameter. The majority of cases occurred as a single mass and were clinically asymptomatic. CT scans revealed all reported cases to possess well-defined nodular lesions, with a good enhancement in the arterial and venous phases following contrast injection. All cases were diagnosed as hepatocellular carcinoma, focal nodular hyperplasia or cavernous hemangioma prior to pathological examination.

These studies suggest that the production of AFP originates from hepatic hemangioma, implying that certain hepatic cavernous hemangioma may possess the ability to synthesize and secrete AFP. For the present patient, the serum AFP level decreased rapidly following hepatectomy. With a 20-year history of chronic hepatitis and an elevated level of tumor markers, the current case prompts consideration of cancer stem cells, which exhibit distinct proliferative and differentiative capacity (16). Although this patient possessed a 20-year history of chronic hepatitis B, which could be a possible reason for elevated serum AFP, the existence of cancer stem cells should not be excluded. According to certain studies into cancer stem cells, it is likely that AFP, CEA and CA199 were produced by cancer stem cells in the hemangioma during development (17–19). The history of chronic hepatitis could concurrently promote the appearance of hepatic cancer stem cells (20–22).

Once a liver lesion with elevated AFP level is found, it is important to differentiate whether the liver lesion is HCC or not. The treatment choice should be based on the nature and extent of disease, so that surgery depends on the surgical risk and the benefit.

In conclusion, clinicians should be aware that there are certain tumors besides HCC and endodermal sinus tumors, such as hepatic cavernous hemangioma, that may produce AFP in adults.

## Figures and Tables

**Table I:** Result of laboratory tests.

Laboratory test	On admission	Post-operative week four	Reference range
Alanine aminotransferase (U/l)	300↑	35	1–40
Aspartate aminotransferase (U/l)	390↑	91↑	1–37
Fasting plasma glucose (mmol/l)	4.6	4.9	2.9–6.0
Urea (mmol/l)	4.4	12.1	2.9–8.6
Serum creatinine (μmol/l)	73	102	53–115
Lactate dehydrogenase (U/ml)	256↑	120↓	114–240
Gamma-glutamyltransferase (U/l)	323↑	101↓	2–50
Alkaline phosphatase (U/l)	98	71	0–110
Total protein (g/l)	76.4	42.3	64.0–87.0
Albumin (g/l)	37.5	32.4	35.0–50.0
Globulin (g/l)	38.9	9.9↓	20.0–32.0
Direct bilirubin (μmol/l)	19.7↑	29.3↑	0.5–7.0
Indirect bilirubin (μmol/l)	25↑	25.2↑	3.0–15.0
Sodium (mmol/l)	139	129	135–145
Potassium (mmol/l)	4.16	4.3	3.5–5.3
Hemoglobin (g/l)	169↑	140	120–160
Hematocrit (proportion of 1.0)	0.494↑	0.28	0.110–0.280
White blood cells (×10^9^/l)	5.19	9.19	4.00–10.00
Platelets (×10^9^/l)	141	288	100–300
Mean corpuscular volume (fl)	96.5↑	95.9↑	82–95
Markers of viral hepatitis			
Hepatitis B surface antigen	+		
Antibody to hepatitis B surface antigen	−		
Antibody to hepatitis C virus	−		
Hepatitis B e-antigen	+		
Antibody to hepatitis B e-antigen	+		

↑ denotes a result higher than the reference range and ↓ denotes a result lower than the reference range.

**Table II:** Serum level of tumor markers.

Tumor marker	On admission	Two days after operation	One week after operation	Two weeks after operation	Reference range
AFP (ug/l)	371.51↑	180	111	24.45	0.00–20.00
CEA (ug/l)	5.59↑				0.00–5.00
CA199 (U/ml)	39.74↑				0.00–35.00
CA125 (U/ml)	14.20				0.00–35.00

↑ denotes a result higher than the reference range and ↓ denotes a result lower than the reference range. AFP, α-fetoprotein; CEA, carcinoembryonic antigen; CA199, carbohydrate antigen 199; CA125, carbohydrate antigen 125.
